# A non-binding surface demonstrates increased thrombogenicity under plasma-free conditions

**DOI:** 10.1038/s41598-025-06636-z

**Published:** 2025-07-02

**Authors:** Cezary Watala, Boguslawa Luzak, Tomasz Przygodzki, Magdalena Boncler

**Affiliations:** Department of Haemostasis and Haemostatic Disorders, Chair of Biomedical Sciences, Medical University of Lodz, ul. Mazowiecka 6/8, Lodz, 92-215 Poland

**Keywords:** Blood platelets, Platelet activation, Cell adhesion, Platelet aggregation, Polypropylenes, Polystyrenes, Medical research, Materials science

## Abstract

Although the commercially available, non-binding microplate is characterised as a low-fouling material, its suitability for the study of platelet function in basic research has yet to be determined. This study compares the non-binding microplate with microplates made of polypropylene (PP) and polystyrene (PS) in terms of thrombogenicity. The thrombogenicity of the non-binding surface was evaluated using several platelet assays performed on platelet-rich plasma (PRP) and suspensions of isolated platelets obtained from healthy donors. In PRP, the non-binding surface exhibited similar or lower thrombogenicity than the PP and PS surfaces. However, with isolated platelets, it exhibited similar or higher thrombogenicity. A clear asymmetry in platelet adhesion to the polymers was observed depending on the presence of plasma components. Adhesion was barely detectable for PRP on the non-binding surface and for isolated platelets on the PS and PP surfaces. Conversely, pronounced platelet deposition was observed for PRP on the PP and PS surfaces, and for isolated platelets on the non-binding surface. These observations suggest that platelets may interact directly with functional groups on the non-binding surface. Consequently, the non-binding surface could serve as an alternative to conventional polymeric surfaces in the in vitro platelet assays, when plasma is present.

**Supplementary Information:**

The online version contains supplementary material available at 10.1038/s41598-025-06636-z.

## Introduction

The search for an ideal biocompatible material for medical applications has led to the development of materials that are highly resistant to non-specific protein adsorption and platelet deposition. Experimental data has shown that incorporating polyethylene oxide/polyethylene glycol (PEO/PEG), poly(hydroxyethyl methacrylate) (pHEMA), hyperbranched polyglycerol (HPG) or zwitterionic molecules (e.g. poly(carboxybetaine acrylamide), or polyCBAA) into the polymer composition enhances its non-fouling properties^1^. Some non-fouling polymer surfaces are commercially available in the form of 96-well microplates, which leading companies in the laboratory equipment market recommend for use in homogeneous assays. This is because they have been shown to significantly reduce the binding of proteins and nucleic acids to the polymer and effectively inhibit the adhesion of epithelial cells (e.g. Madin-Darby canine kidney cells, MDCK cells). However, the suitability of the 96-well non-binding microplate for studying platelet function remains to be determined.

In vitro platelet assays are most commonly performed using disposable polypropylene (PP) tubes, pipette tips or Pasteur pipettes. These consumables have a variety of uses, including blood collection, preparing platelet-rich plasma (PRP), transferring and measuring samples^2^. Microplate-based assays for platelet adhesion and aggregation use 96-well polystyrene (PS) microplates that are typically pre-coated with proteins. This is particularly the case in platelet adhesion studies^3–5^. Standard flow cytometric platelet studies and impedance whole blood platelet aggregometry are also performed in PS tubes^6,7^. In the latter case, the measurement is performed under mixing conditions and is based on platelet adhesion to metal electrodes embedded in the test cuvette^8^. Some platelet assays, such as optical aggregometry (LTA, light transmission aggregometry), are carried out in silicone-coated glass cuvettes to protect platelets from activation. Pure glass tends to form clots or thrombi, when in contact with blood and is therefore used as a positive control in thrombogenicity testing of biomaterials^9,10^.

For assays where avoiding protein-solid surface interactions is essential, non-binding microplates have been suggested as a better option than traditional PP and PS microplates^11^. This suggests that the non-binding surface could be used instead of PP for testing platelet function in vitro. Our study therefore aimed to investigate the usefulness of the non-binding surface for this purpose. Experiments were performed on PRP and on platelets suspended in Tyrode’s buffer, both after contact with the non-binding microplate and with reference surfaces (PP and PS microplates). Platelet reactivity was assessed by measuring platelet activation, adhesion, secretion and aggregation. Observations from platelet function tests were supplemented by microscopic analysis of platelet deposition on polymeric surfaces.

## Results

### Characteristics of the study group

The study group was characterised by haematological and biochemical parameters (white blood cell count, WBC; red blood cell count, RBC; haemoglobin, HGB; hematocrit, HCT; mean corpuscular volume, MCV; platelet count (PLT); mean platelet volume, MPV; C-reactive protein; CRP). The results presented in Table 1 show that all the indices considered in this study were within the reference ranges for both men and women.

**Table 1:** Selected haematological and biochemical variables measured in the examined participants.

Parameter	Men (*n* = 7)	Reference range	Women (*n* = 18)	Reference range
Age (years)	40 ± 10		42 ± 14	
WBC count (x 10^3^/µl)	5.5 ± 1.6	4.0‒11.0	6.3 ± 1.0	4.0‒11.0
RBC count (x 10^6^/µl)	5.0 ± 0.2	4.2‒6.1	4.5 ± 0.2	3.7‒5.1
HGB (g/dl)	15.2 ± 0.4	14.0‒18.0	13.3 ± 0.6	12.0‒16.0
HCT (%)	43.8 ± 1.4	40.0‒55.0	39.5 ± 1.8	36.0‒48.0
MCV (fl.)	87.3 ± 1.0	80.0‒98.0	87.3 ± 3.1	80.0‒98.0
PLT (x 10^3^/µl)	237 ± 62	150‒400	268 ± 37	150‒400
MPV (fl.)	10.5 ± 1.7	8.0‒12.0	10.3 ± 0.9	8.0‒12.0
CRP (mg/l)	1.2 ± 0.6	0‒5	1.7 ± 1.5	0‒5

Data are shown as mean ± SD. The reference ranges intervals for hematological and biochemical blood parameters were established by the local laboratory.

### Changes in P-selectin expression and fibrinogen binding after exposure of platelets to the non-binding surface

**Fig. 1:**
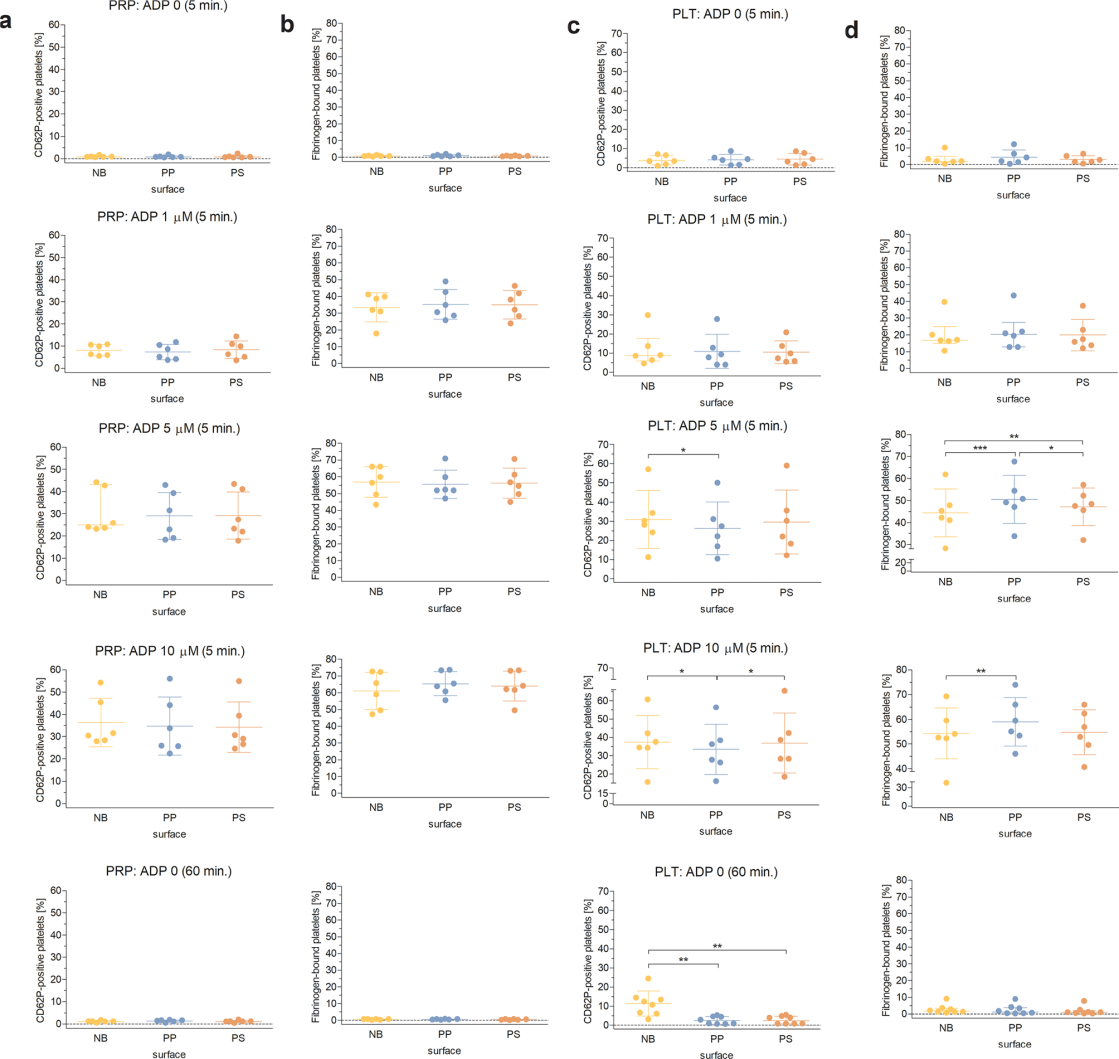
Changes in platelet activation after exposure to polymer surfaces. Platelet activation was assessed by measuring P-selectin expression (a, c) and fibrinogen binding (b, d) after 5 and 60 min exposure of platelet-rich plasma (PRP: a, b) or isolated platelets (PLT: c, d) to the non-binding (NB), polypropylene (PP) and polystyrene (PS) surfaces. Measurements were performed in response to different concentrations of ADP (0, 1, 5, 10 µM). Data with normal distribution are presented as mean ± SD, otherwise data are presented as median with interquartile range, *n* = 6‒8. Statistical significance of the differences was assessed using either a repeated-measures ANOVA or a Friedman test. Due to low statistical power, the bootstrap method, involving 10,000 iterations was applied to the significant comparisons. Statistically significant differences are indicated by asterisks (*P < 0.05, **P < 0.01, ***P < 0.001).

The fraction of P-selectin-positive platelets in unstimulated PRP samples after incubation for 5 and 60 min was 0.9 ± 0.4% and 1.2 ± 0.4% for the non-binding surface (NB), 0.9 ± 0.5% and 1.3 ± 0.5% for polypropylene (PP) and 0.9 ± 0.7% and 1.1 ± 0.5% for polystyrene (PS), respectively; no significant differences in P-selectin expression were observed between the surfaces. Incubation of PRP on polymer surfaces in the presence of 1, 5, and 10 µM ADP for 5 min resulted in a substantial increase in P-selectin expression (up to 36.3 ± 10.9% of CD62-positive platelets for NB at the highest ADP concentration), although the values did not differ between the groups (Fig. 1a). Similar observations were made in PRP samples when fibrinogen binding was measured. Indeed, in unstimulated platelets, the fraction of fibrinogen-bound cells after a 5 and 60 min incubation on polymeric surfaces was low (0.9 ± 0.4% and 0.5 ± 0.2% for NB, 1.1 ± 0.6% and 0.5 ± 0.2% for PP, and 0.8 ± 0.2% and 0.4 ± 0.2% for PS, respectively), and markedly increased after stimulation with ADP (up to 61.1 ± 11.1% of fibrinogen-bound platelets for NB at the highest ADP concentration) (Fig. 1b). The differences in the proportion of fibrinogen-bound platelets measured in PRP between the surfaces were not significantly different, regardless of the model.

In isolated platelets, the percentage of CD62-positive cells was 3.9 ± 2.4% and 11.4 ± 6.6% for NB, 4.2 ± 2.7% and 2.5 ± 2.0% for PP, and 4.5 ± 3.0% and 2.6 ± 2.1% for PS, after 5 and 60 min of incubation, respectively. Furthermore, P-selectin expression was over 4 times higher in platelets exposed to the non-binding surface for 60 min than in platelets exposed to PP or PS (*P* < 0.01). Significant increases in P-selectin expression were also observed for the non-binding surface compared to PP in platelets stimulated with 5 µM ADP and 10 µM ADP (*P* < 0.05), as well as for PS compared to PP in platelets stimulated with 10 µM ADP (*P* < 0.05) (Fig. 1c).

The percentage of fibrinogen-bound cells in suspensions of isolated platelets after a 5- and 60-min incubation on polymeric surfaces was 3.3 ± 3.5% and 2.7 ± 2.7% for NB, 4.5 ± 4.3% and 2.5 ± 3.0% and for PP, and 3.1 ± 2.2% and 1.8 ± 2.5% for PS, respectively; no significant differences in the proportion of fibrinogen-bound platelets were observed between the surfaces. The percentage of fibrinogen-bound platelets was higher on ADP stimulation (up to 54.3 ± 10.3% for NB at 10 µM ADP). Surprisingly, the proportion of fibrinogen-bound platelets measured on polypropylene was significantly higher than on other surfaces, including the non-binding surface at both 5 µM and 10 µM ADP (an increase of 1.1-fold, *P* < 0.001 and *P* < 0.01, respectively), as well as on polystyrene in platelets stimulated with 10 µM ADP (an increase of 1.1-fold, *P* < 0.05). Furthermore, the percentage of fibrinogen-bound platelets measured on polystyrene after stimulation with 5 µM ADP was notably higher than on the non-binding surface (*P* < 0.01) (Fig. 1d). A summary of the main results is provided in Supplementary Table S1.

### Changes in soluble P-selectin levels following exposure of platelets to the non-binding surface

**Fig. 2:**
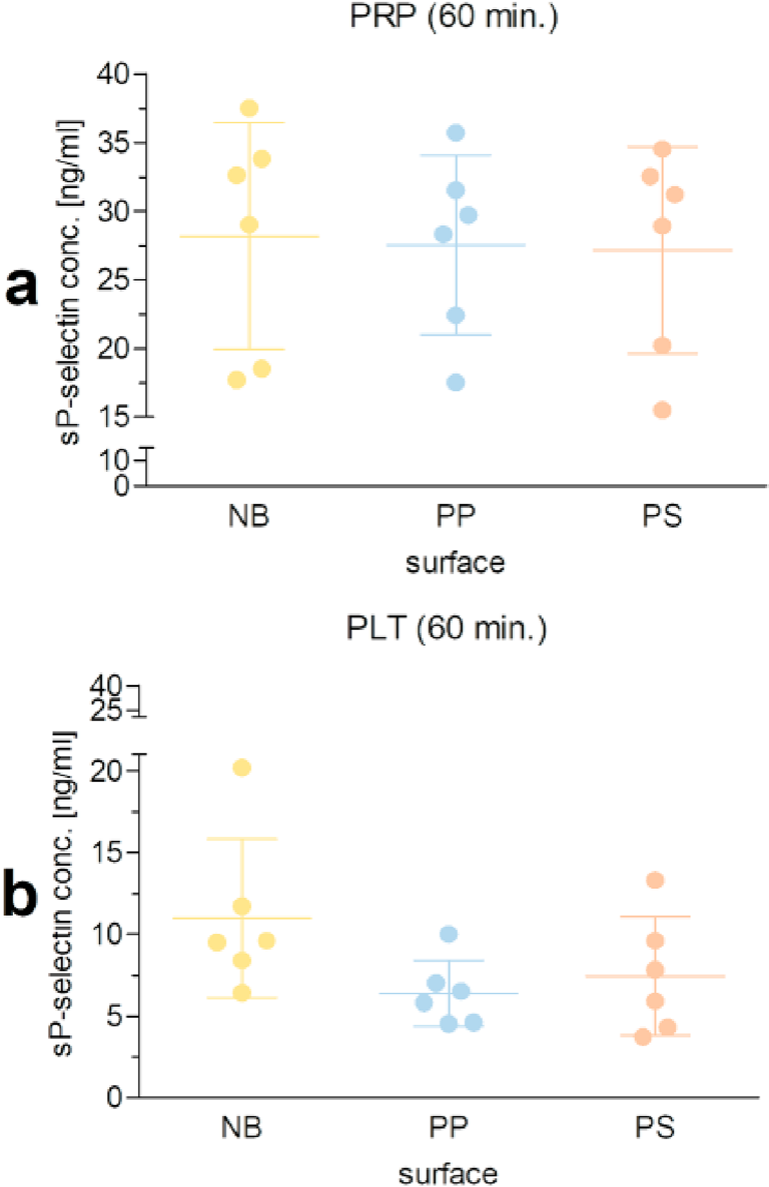
Soluble P-selectin concentration following exposure of blood platelets to polymer surfaces. Data are presented as mean ± SD, *n* = 6. Levels of sP-selectin were determined in plasma prepared from platelet-rich plasma (PRP: a) and platelet supernatants obtained from suspensions of isolated platelets (PLT: b) after 60 min incubation of the samples on the non-binding (NB), polypropylene (PP), and polystyrene (PS) surfaces. Differences between the groups were analysed by RM ANOVA with Tukey’s post-hoc test.

Changes in soluble P-selectin (sP-selectin) were analysed in a model where platelets were exposed to polymer surfaces for 60 min. The concentration of sP-selectin measured in plasma was similar for the surfaces studied, but approximately 2-4-times higher than that measured in isolated platelets (Fig. 2a). sP-selectin levels in isolated platelets exposed to the non-binding surface, were 1.7-fold higher than those of PP and 1.5-fold higher than those of PS, but these differences were not statistically significant, as was the case when PP and PS were compared (Fig. 2b). In addition, when the data collected for each surface were pooled, a positive correlation was observed between the proportion of CD62-positive platelets and the sP-selectin concentration (R_s_=0.727, *P* < 0.001 for platelet supernatants vs. R_s_=0.423, *P* = 0.080 for plasma).

### Effect of the non-binding surface on platelet adhesion in colorimetric assay

Platelet deposition on polymer surfaces was evaluated in unstimulated platelets and ADP-stimulated platelets. Overall, ADP (10 µM) enhanced platelet deposition on polymer surfaces, but significant differences between unstimulated and stimulated cells were found only for PRP, for the non-binding surface (*P* = 0.004), polypropylene (*P* < 0.001) and polystyrene (*P* < 0.05).

**Fig. 3:**
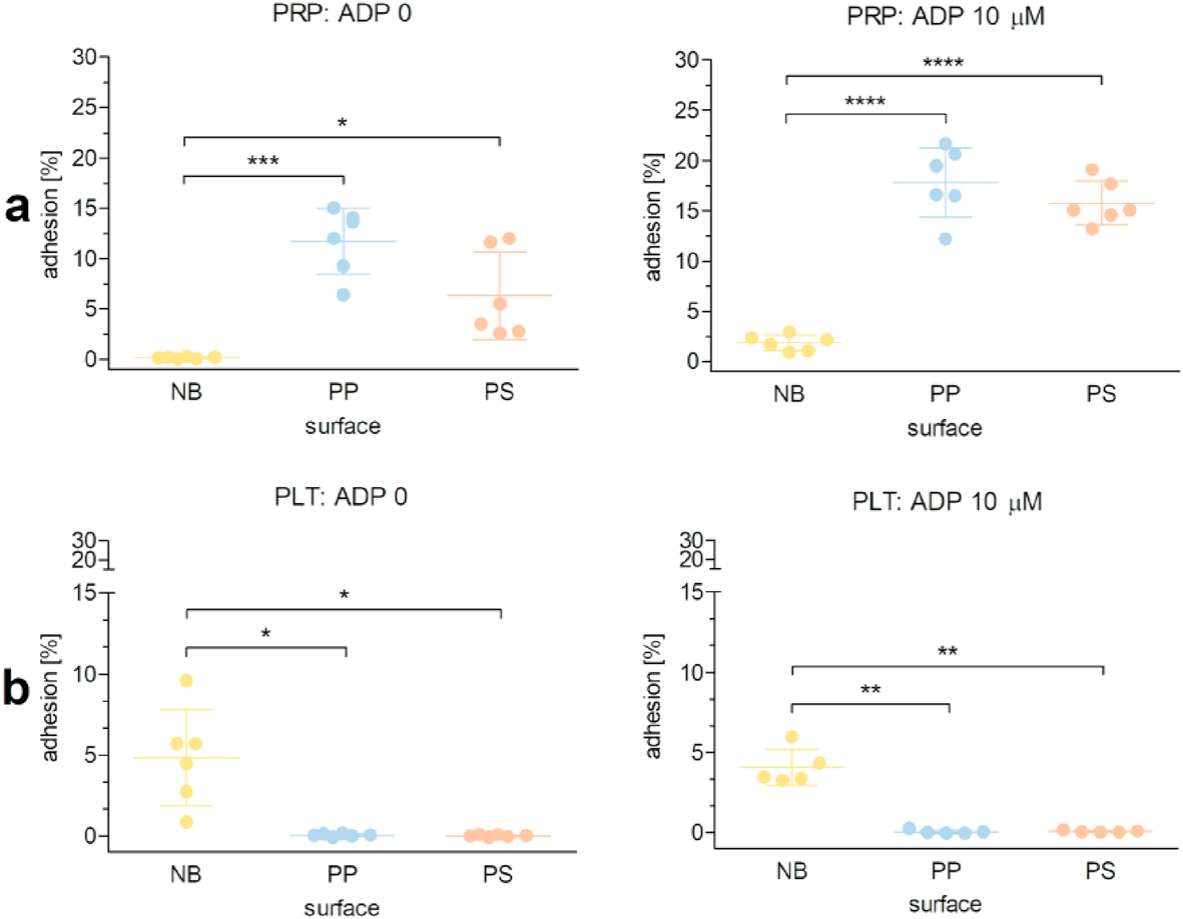
Platelet adhesion on polymer surfaces quantified by colorimetric assay. Platelet adsorption to the non-binding (NB), polypropylene (PP), and polystyrene (PS) surfaces was evaluated in platelet-rich plasma (PRP: a) and suspensions of isolated platelets (PLT: b), which were incubated with and without 10 µM ADP. Data are presented as mean ± SD, *n* = 5‒6. Differences between the groups were assessed using RM ANOVA with Tukey’s post-hoc test. Statistically significant differences are indicated by asterisks (*P < 0.05, **P < 0.01, ***P < 0.001, ****P < 0.0001).

In experiments with PRP, platelet adhesion to the non-binding surface was barely detectable in the absence of ADP (0.2 ± 0.1%) or very low in the presence of ADP (1.9 ± 0.8%). In addition, significantly fewer platelets adhered to the non-binding surface than to PP (*P* < 0.001 and *P* = 0.0001 in unstimulated and ADP-stimulated cells, respectively) and PS (*P* < 0.05 and *P* < 0.0001 in unstimulated and ADP-stimulated cells, respectively). However, no significant differences were observed between platelet adhesion to PP and PS (Fig. 3a).

Experiments with suspensions of isolated platelets showed a different pattern of platelet adhesion to polymer surfaces. Deposition of isolated platelets on the non-binding surface was evident in the absence or presence of ADP (4.8 ± 3.0% and 5.2 ± 3.0%, respectively), and was much higher compared with PP (*P* < 0.05 and *P* < 0.01, resp. in unstimulated and ADP-stimulated platelets) and PS (*P* < 0.05 and *P* < 0.01, resp. in unstimulated and ADP-stimulated platelets). There were no significant differences between platelet adhesion to PP and PS (Fig. 3b). Supplementary Table S1 summarizes the main results.

### Image-based analysis of platelet adhesion on the non-binding surface

**Fig. 4:**
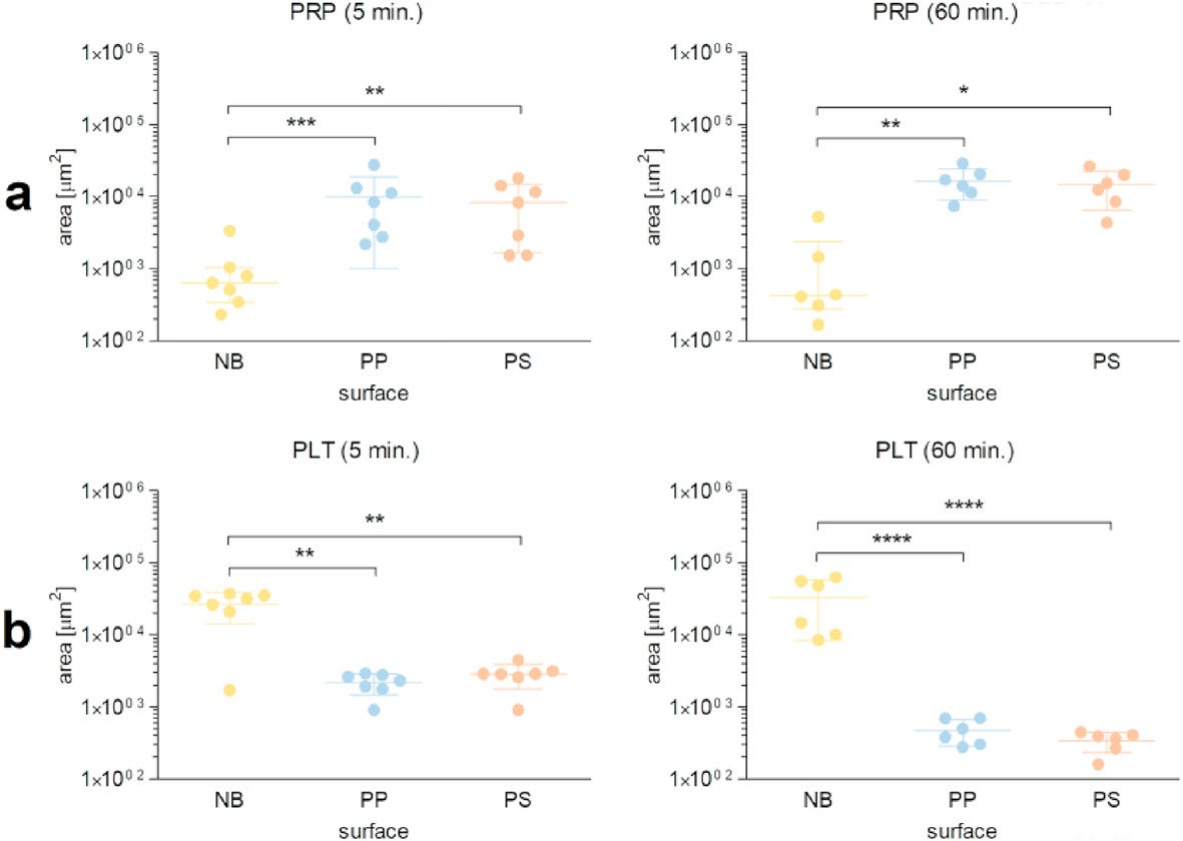
Quantification of platelet covered area on polymer surfaces. The platelet covered area was assessed after preincubation of platelet-rich plasma (PRP: a) and suspensions of isolated platelets (PLT: b) for 5–60 min at 37 °C on the non-binding (NB), polypropylene (PP), and polystyrene (PS) surfaces. Data are presented as mean ± SD, except for NB surface in PRP (5 and 60 min), which are presented as median with interquartile range, *n* = 6‒7. Differences between the groups were assessed using RM ANOVA with Tukey’s post-hoc test. Data violating assumptions were transformed (natural log) for analysis. Statistically significant differences are indicated by asterisks (*P < 0.05, **P < 0.01, ***P < 0.001, ****P < 0.0001).

Figure 4 summarizes data obtained by microscopic evaluation of platelet adhesion quantified as the total area covered by platelets in plasma (PRP) or isolated platelets after 5 and 60 min exposure to polymer surfaces.

**Fig. 5:**
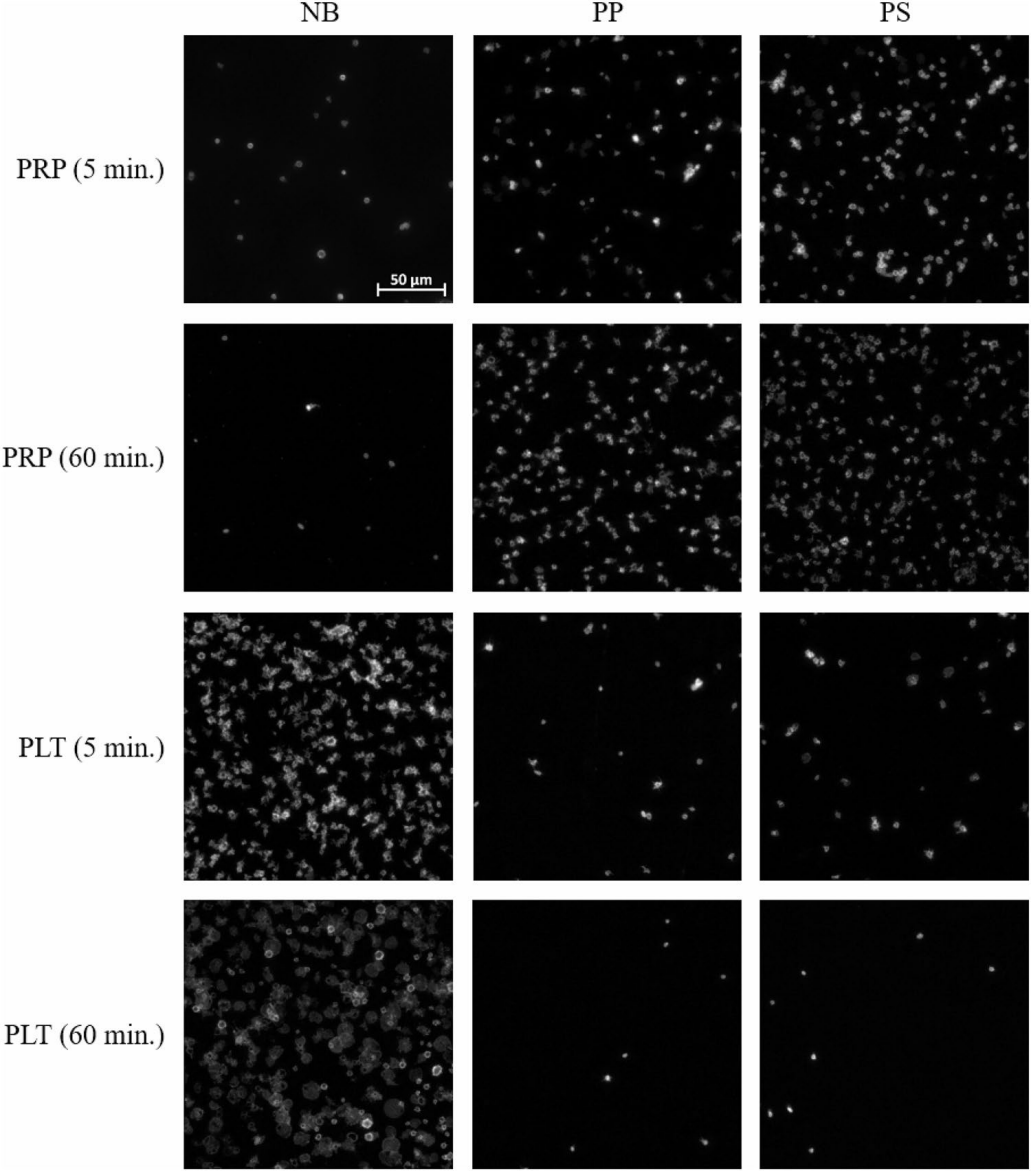
Representative images of adherent platelets after exposure to polymeric surfaces. Experiments were performed in platelet-rich plasma (PRP) and suspensions of isolated platelets (PLT), which were incubated for 5–60 min at 37 °C on the non-binding (NB), polypropylene (PP), and polystyrene (PS) surfaces. Images were taken at 40 × magnification in 6‒7 independent experiments. Further details can be found in Materials and Methods.

For PRP, the platelet-covered area for the non-binding surface was significantly lower than that for polypropylene (a 10-fold (*P* < 0.001), and 12-fold (*P* < 0.01) reduction after 5 and 60 min exposure, respectively) and polystyrene (an 8-fold (*P* < 0.01), and 11-fold (*P* < 0.05) reduction after 5 and 60 min incubation) (Fig. 4a). In contrast, in suspensions of isolated platelets, the platelet-covered area for the non-binding surface was significantly higher than that for polypropylene (a 12-fold (*P* < 0.01) and 70-fold (*P* < 0.0001) increase after 5 and 60 min exposure, respectively) and polystyrene (a 10-fold (*P* < 0.01) and 99-fold (*P* < 0.0001) increase after 5 and 60 min exposure, respectively) (Fig. 4b). No significant differences between PP and PS were found in PRP or in suspensions of isolated platelets. The main results are summarized in Supplementary Table S1. Representative fluorescence microscopic images of platelets in plasma (PRP) and buffer (isolated platelets) on polymer surfaces are shown in Fig. 5.

### Comparison of platelet aggregation on polymer surfaces

**Fig. 6:**
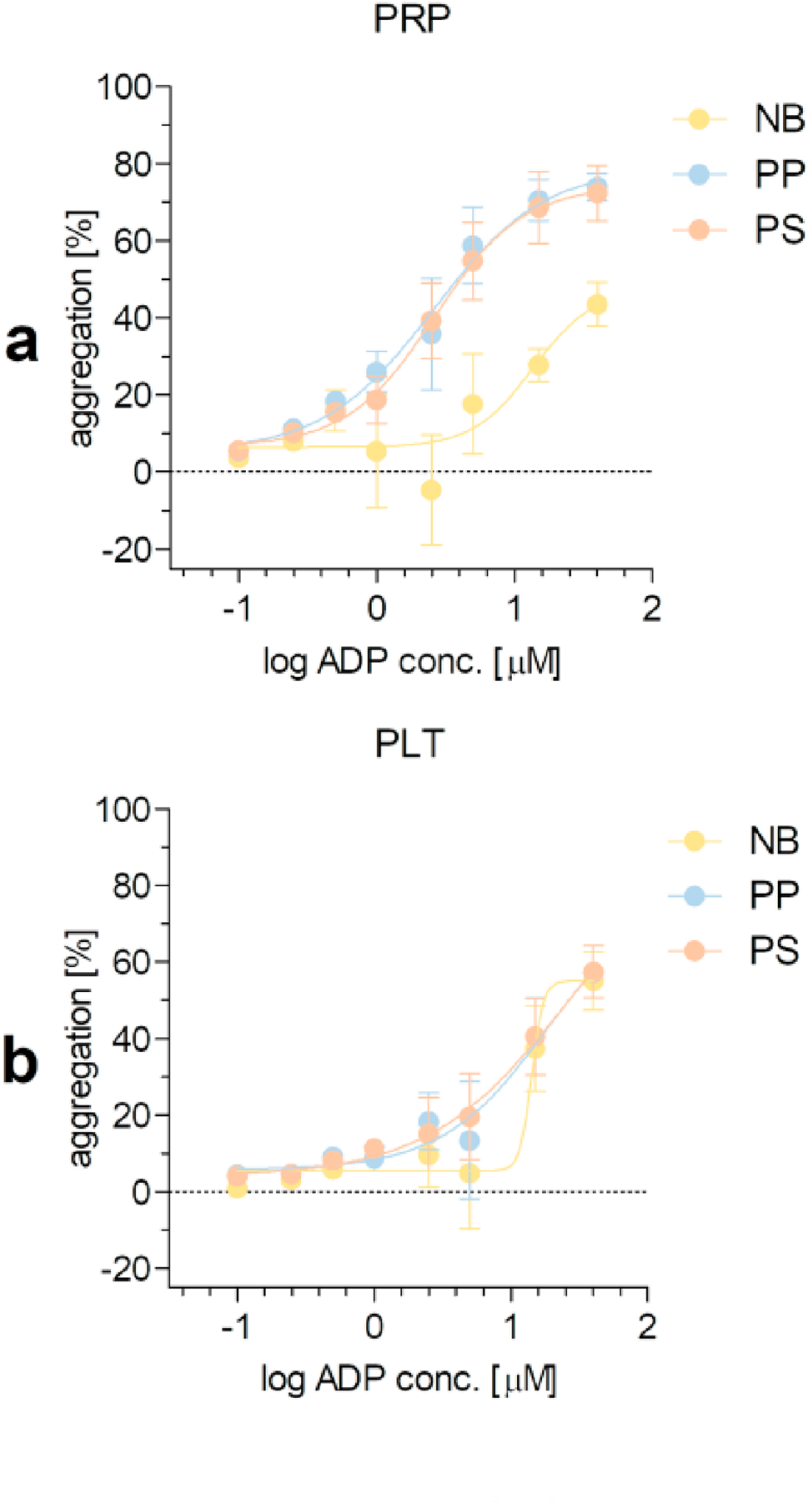
ADP-induced platelet aggregation recorded in a 96-well microplate reader. Data shown as mean ± SE, *n* = 6. Measurements were performed in PRP (a) and suspensions of isolated platelets (PLT, b) on the non-binding (NB), polypropylene (PP), and polystyrene (PS) surfaces in response to 0.1‒40 µM ADP. Differences between curves were tested by F-test. Statistically significant differences were found for PRP (NB vs. PP and NB vs. PS, *P* < 0.0001).

The mean EC50 values for ADP in the aggregation experiments performed in PRP were as follows: 13.6 ± 2.3 µM for NB, 2.6 ± 1.5 µM for PP, and 2.6 ± 1.4 µM for PS. Furthermore, the aggregation curves obtained for PP and PS were similar, but significantly different from those obtained for the non-binding surface (*P* < 0.0001 vs. PP and PS) (Fig. 6a).

When aggregation was performed on isolated platelets, the EC50 values for ADP in this assay were calculated for PP and PS (18.7 ± 5.1 µM for PP and 25.1 ± 17.1 µM for PS), whereas they were estimated for the non-binding surface (~ 14.2 µM). Furthermore, the aggregation curves for NB, PP and PS were not found to be significantly different, although the shape of the curve obtained for NB was different from the shape of the curve for PP and PS (Fig. 6b).

## Discussion

Conventional polypropylene and polystyrene surfaces are widely used in basic and medical research. A lesser known labware that has not yet been evaluated for thrombogenicity is a commercial non-binding surface that has low protein, DNA and peptide binding properties, which significantly improves data quality in biochemical assays. Non-binding potential is achieved by chemically modifying the polystyrene surface to form a hydrate layer that prevents molecules from binding to it. This process involves covalently attaching functional groups to the polystyrene^11,12^. In this report, we describe the effect of the non-binding surface on human blood platelet activation, adhesion, secretion, and aggregation and compare the results with those of PS and PP surfaces. All of these surfaces have applications in biochemical testing. The proposed experimental approach, based on the analysis of platelet function at two time points in both, PRP and isolated platelets, allowed the evaluation of potential differences between surfaces after short and long contact of platelets with the polymer surface and to gain insight into whether plasma components affect the results. We have shown the significant influence of the non-binding surface on platelet function in the absence of plasma compared to PS or PP; the greatest differences between the surfaces studied were found in platelet adhesion and visualisation assays (Supplementary Table S1).

Irrespective of exposure time, experimental model (w/wo agonist) and marker selected (platelet P-selectin, fibrinogen binding, soluble P-selectin), fully comparable results were obtained between all surfaces when assessing platelet activation in platelet-rich plasma. Thus, based on the agreement between the results of the platelet activation assays conducted on different surfaces, it can be concluded that the non-binding surface can be used instead of the PP and PS surfaces to analyze platelet activation in the presence of blood plasma. This also implies that in other assays the non-binding surface may differ from the reference surfaces in terms of thrombogenicity, which appears to be strongly influenced by plasma components. Indeed, the results of platelet adhesion and aggregation on PRP showed that the non-binding surface was the least thrombogenic material compared to the other surfaces, PS and PP. This was demonstrated by a significantly lower spontaneous and ADP-induced platelet deposition on the non-binding surface compared to PP and PS (Figs. 3a and 4a) and a reduced platelet reactivity on the non-binding surface compared to PP and PS in the ADP-induced platelet aggregation assay (Fig. 6a), as evidenced by a > 5-fold higher EC50 value for ADP calculated for the non-binding surface compared to the EC50 values obtained for the other surfaces. These results are consistent with a previous study on PRP, in which the non-binding surface was found to have a significantly lower capacity for platelet adhesion and protein adsorption than the other polystyrenes: untreated PS and high-binding PS^13^.

On the other hand, in the absence of plasma (experiments with isolated platelets), the non-binding surface was shown to be equally or more thrombogenic than other surfaces. The EC50 values for ADP-induced aggregation using isolated platelets did not differ between the surfaces used, although it should be noted that the EC50 for NB could only be estimated, not calculated, as the model could not be fully defined by the available data. Furthermore, the aggregatory effect of ADP recorded on the reference surfaces was partial and did not reach a plateau, suggesting that isolated platelets may be more susceptible to aggregation when exposed to the non-binding surface as compared to the PP and PS (Fig. 6b). The much higher thrombogenicity of the non-binding surface compared to the reference surfaces was demonstrated by analysing platelet deposition on the polymer microplates (colorimetric and microscopic study) (Figs. 3b and 4b). The non-binding surface was also more thrombogenic when platelet activation was assessed in isolated platelets stimulated with 5 or 10 µM aDP, and after prolonged contact of platelets with the artificial surface. This was evidenced by significantly higher P-selectin expression in platelets exposed to the non-binding surface than to PP or PS. Furthermore, we observed a strong positive correlation between P-selectin expression and sP-selectin levels in isolated platelets, but surprisingly we did not find significant differences in sP-selectin levels between the non-binding surface and other surfaces. These differences were substantial (≥ 1.5-fold), so we can speculate that the smaller sample size for sP-selectin measurements compared to P-selectin expression led to a failure to detect significant changes between surfaces. Interestingly, while we have shown a dependence between the percentage of platelets expressing CD62P and sP-selectin concentration in isolated platelets, no such relationship was observed in PRP. The available literature is inconsistent on this point. Some reports show a positive association (R_s_=0.307 in patients with peripheral artery disease), others suggest a negative association (R_s_=-0.257 in patients with stable congestive heart failure)^14,15^, and still others show no association between these platelet indices (studies in patients with peripheral artery disease, atrial fibrillation, congestive heart failure and controls)^16,17^. More striking was the observation that the sP-selectin levels in plasma, which averaged 30 ng/ml, were lower than previously reported levels of between 40 and 80 ng/ml found in citrated plasma from healthy individuals^16,18,19^. This difference may be explained by the lower number of platelets in PRP from which plasma was obtained for sP-selectin measurements (PRP was standardised for platelet count), and blood platelets are known to be an important source of sP-selectin.

Interesting results were obtained in the platelet adhesion experiments, where the asymmetry of the platelet response depending on the presence of plasma was remarkable. Adhesion was barely detectable for PRP on the non-binding surface and for isolated platelets on PS and PP, whereas pronounced platelet deposition was observed for PRP on reference surfaces and for isolated platelets on the non-binding surface, with the highest platelet density after 60 min exposure of the samples to the artificial materials (Fig. 6). These observations may suggest a direct interaction of platelets with functional groups on the non-binding surface, which may occur in the absence of plasma, particularly those proteins that promote platelet adhesion. It is conceivable that in the presence of plasma, the non-binding surface, unlike the reference surfaces, drastically reduces the adsorption of proteins (including those that mediate platelet adhesion), resulting in very limited platelet adhesion, whereas in the absence of plasma components, especially those that mediate platelet adhesion, platelets can bind directly to the artificial surface (in our experiments isolated platelets were suspended in Tyrode’s buffer containing trace amounts of HSA). This is likely to occur because the non-binding surface exhibits features of the polyethylene oxide (PEO) polymer, also known as polyethylene glycol (PEG). It has been demonstrated that there are weak hydrophobic interactions between this polymer and model proteins, such as bovine serum albumin and lysozyme^20^. It is also possible to speculate that platelet-derived proteins contribute to platelet adhesion to non-binding surfaces. During activation, platelets release proteins such as fibrinogen and von Willebrand factor from their alpha granules. These proteins may bind to and/or adsorb onto the non-binding surface, thereby promoting platelet adhesion in the absence of plasma.

In conclusion, our study has evaluated the usefulness of a non-binding surface for platelet testing under laboratory conditions using several different assays. The thrombogenicity test results for platelet-rich plasma obtained using a non-binding microplate were comparable to, or even better than those obtained using polypropylene or polystyrene microplates. This suggests that the former could be used for in vitro platelet studies involving blood plasma. However, in experiments involving isolated platelets, the non-binding microplate exhibited similar or greater thrombogenicity than the polypropylene and polystyrene microplates. Therefore, it is not recommended for use under plasma-free conditions. It is important to note that these results relate to artificial surfaces from a single source, so conclusions should be drawn with caution.

## Materials and methods

### Reagents

Mouse anti-human CD62P/PE (clone AC1.2), mouse IgG1/PE isotype control, Cellfix, and blood collection tubes containing a buffered sodium citrate solution (0.109 M) were purchased from Becton-Dickinson (Franklin Lakes, NJ, USA). Fibrinogen from human plasma, Oregon Green 488 Conjugate (OG-fibrinogen) were purchased from Invitrogen (Carlsbad, CA, USA). PE-conjugated anti-human CD41 antibodies (clone 5B12) were from Agilent Technologies (Santa Clara, CA, USA). Human P-Selectin/CD62P immunoassay was from Bio-Techne (Abingdon, UK).

Prostaglandin E_1_ (PGE_1_), human serum albumin, apyrase, fibrinogen from human plasma, adenosine 5’-diphosphate sodium salt (ADP), calcium chloride, p-nitrophenyl phosphate (pNPP), Triton X-100, were from Sigma (St. Louis, MO, USA). Other chemicals, unless otherwise stated, were purchased from Avantor Performance Materials Poland S.A. (Gliwice, Poland). Phosphate-buffered saline (PBS), pH 7.4, was from Corning (Corning, NY, USA). Sodium chloride solution (0.9% in water) was from Glenmark Pharmaceuticals (Warsaw, Poland). S-Monovette^®^ tubes were from Sarstedt (Numbrecht, Germany). Ninety six-well polystyrene flat-bottom ELISA plates (untreated surfaces (PS, #655101) and surfaces treated to prevent protein binding (NB, #655901), and 96-well polypropylene (PP, #655261) flat-bottom ELISA plates were from Greiner Bio-One (Frickenhausen, Germany).

### Participants

Human blood was collected from consecutively recruited healthy volunteers: 7 males and 18 females; mean age 41 ± 13 years. All individuals gave their written informed consent to participate in the study. All subjects confirmed that they had not taken any medication known to affect platelet function (e.g. non-steroidal anti-inflammatory drugs, NSAIDs) for at least two weeks prior to the study. The study was approved by the Human Research Committee of the Medical University of Lodz, Poland (RNN/323/20/KE) and was conducted according to the Declaration of Helsinki guidelines.

### Study design

**Fig. 7:**
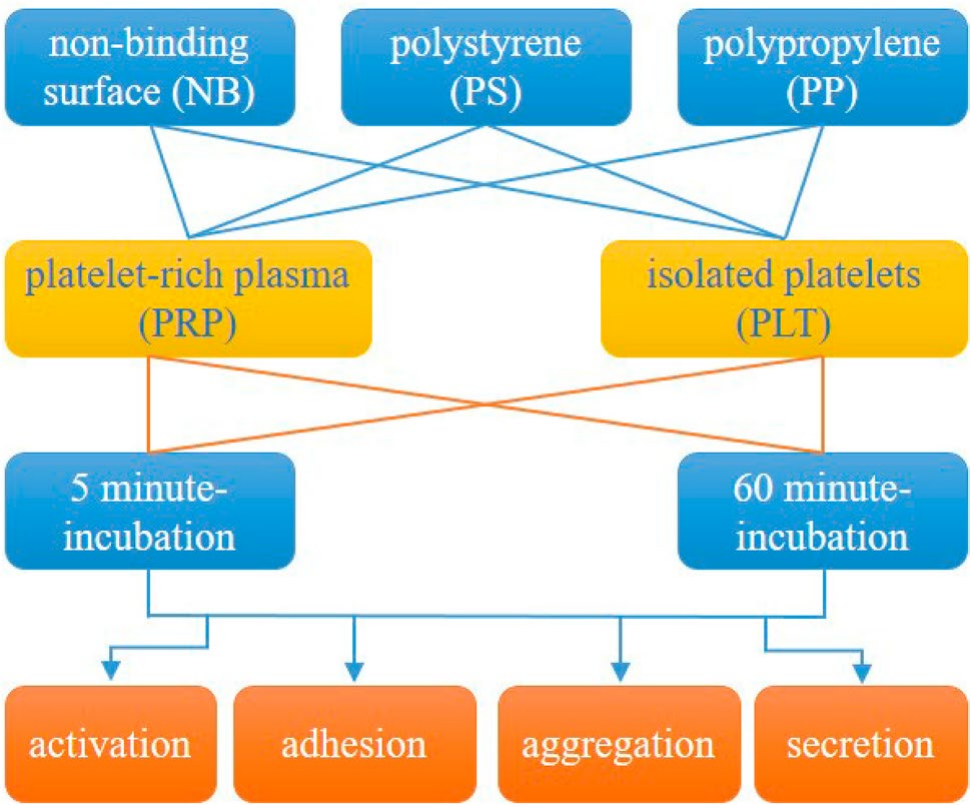
Design of the study.

The thrombogenicity of the non-binding surface was evaluated in several platelet assays (activation, adhesion, aggregation, release reaction) against two reference surfaces: PS and PP. Platelet function tests were performed on either platelet-rich plasma (PRP) or the suspensions of isolated platelets after 5 and 60 min of contact with solid surfaces. Platelet aggregation typically takes a few minutes, whereas platelet adhesion under static conditions is monitored over a longer period of time. Therefore, these tests were performed at only one time point. For most tests, the platelet count was 3 × 10^8^ cells per ml; however some assays required optimisation for platelet density. Platelet adhesion to artificial surfaces was quantified at different cell counts: at 5 × 10^7^ cells per ml in a colorimetric assay^21^, and at 3 × 10^8^ cells per ml (for 5 min incubation) or at 3 × 10^7^ cells per ml (for 60 min incubation) in the microscopic analysis (platelet count was chosen experimentally). All platelet assays were performed within the recommended time after blood collection. A schematic diagram of the study design is shown in Fig. 7.

### Blood collection and processing

Blood samples were taken from fasting participants in the morning between 8.00 and 9.00 a.m. using light tourniquets and short and moderate phlebostasis from the antecubital vein with 19-gauge needles. The first millilitres of blood were collected in EDTA and serum tubes for complete blood count (CBC) and C-reactive protein level. The CBC parameters were measured using an XN-100 haematology analyser (Sysmex, Kobe, Japan), while CRP was measured using an AU700 chemistry analyser (Beckman Coulter, Brea, CA, USA). Blood samples for platelet testing were collected according to the guidelines for performing multi-color flow cytometry in whole blood^22^ and the ISTH recommendations for standardization of light transmission aggregometry^23^. The samples were collected in polypropylene tubes (S-Monovette^®^) prefilled with a sodium citrate solution (0.109 M) at a blood/anticoagulant ratio of 9:1 vol/vol. The tubes were gently inverted several times immediately after blood collection in order to ensure proper mixing with the anticoagulant. Platelet-rich plasma was prepared by centrifugation of the collected blood at 200 × g for 12 min at 37 °C. Platelet-poor plasma (PPP) was obtained by centrifugation of PRP samples at 2000 × g for 15 min at 37 °C. Platelets were isolated according to the described protocols with minor modifications^24–26^. Briefly, platelets were sedimented from PRP by centrifugation (700 × g, 15 min) and washed in Tyrode’s buffer (134 mM NaCl, 12 mM NaHCO_3_, 2.9 mM KCl, 0.34 mM Na_2_HPO_4_, 1 mM MgCl_2_, 10 mM HEPES, 5 mM glucose, pH 7.4) containing PGE_1_ (300 ng/ml). The washed platelets were then resuspended in Tyrode’s buffer containing HSA (0.2%), CaCl_2_ (1 mM), apyrase (0.02 U/ml) and adjusted to the appropriate number of cells. For aggregation experiments, the buffer also contained fibrinogen (0.5 µg/ml) to enhance platelet response to ADP. Platelet count was measured using a XS-800i haematology analyser (Sysmex, Kobe, Japan).

### Measurement of platelet activation markers: expression of P-selectin and fibrinogen binding

P-selectin expression and fibrinogen binding on platelets were quantified by flow cytometry. Platelet activation was measured after 5 and 60 min of cell contact with polymer surfaces (100 µl PRP or suspension of isolated platelets per well of the 96-well plate). For short incubation time, platelet activation was also assessed in the presence of 1, 5, 10 µM ADP. To assess platelet degranulation, the platelet samples were transferred from the wells into polypropylene tubes and fixed with 1% paraformaldehyde (1 h, 37 °C). The samples were then labelled with anti-human CD62/PE antibodies (3 µl per 10 µl fixed sample of PRP and 20 µl of fixed platelet suspension) for 20 min at RT, and diluted with PBS to a final volume of 300 µl. To evaluate the effect of the polymer surface on fibrinogen binding to platelets, cells were maintained in the wells in the presence of OG-fibrinogen (30 µg/ml) for 5 min at 37 °C. After incubation, the supernatants were collected in polypropylene tubes, fixed with 1% paraformaldehyde (1 h, 37 °C), and diluted in PBS for standard cytometric analysis. The fractions of CD62P-positive platelets and OG-positive platelets were determined in FSC/SSC gated population of blood platelets using a FACSCanto II cytometer (Becton-Dickinson, Franklin Lakes, NJ, USA). Ten thousand events per sample were acquired and data were processed using FACS/Diva software (Becton-Dickinson, Franklin Lakes, NJ, USA).

### Determination of soluble P-selectin concentration

Soluble P-selectin (sP-selectin) levels were determined in plasma and supernatants of isolated platelets after incubation of samples on polymer surfaces for 60 min using a sandwich ELISA. Plasma and platelet supernatants were obtained by centrifugation of PRP and isolated platelet suspensions (2000 x g for 20 min) and stored at ‒80 °C until use.

### Study of platelet adhesion

#### Colorimetric assay

Platelet adhesion to uncoated microplates was measured quantitatively as previously described^27^ using PRP and suspensions of isolated platelets. Platelets in plasma (PRP) were adjusted to 3 × 10^8^ cells per ml with PPP and then diluted 6-fold to 5 × 10^7^ cells per ml with saline. Suspensions of isolated platelets were adjusted to 5 × 10^7^ cells per ml with Tyrode’s buffer. Platelets were allowed to adhere for 1 h at 37 °C under static conditions in the absence or presence of 10 µM ADP. The microplates were then carefully washed three times with PBS and the wells were filled with lysis buffer (0.1 M citrate buffer, pH 5.4, containing 1 mg/ml p-nitrophenyl phosphate and 0.1% Triton X-100). After incubation for 1 h at 37 °C, the reaction was stopped with 2 M sodium hydroxide. The p-nitrophenol generated by the reaction was measured using a VICTOR^™^ X4 microplate reader (Perkin Elmer, Waltham, MA, USA) at 405 nm.

#### Imaging and quantification of adhered blood platelets

One hundred microlitres of PRP or isolated platelets were added to the polymer surface wells and incubated under static conditions at 37 °C for 5 or 60 min. The wells were then washed with PBS and the samples fixed with 1% paraformaldehyde (PFA) for 30 min at 37 °C. After three washes, adherent cells were stained with anti-human CD41/PE diluted 1:5 in PBS for 30 min at RT in the dark. After staining, samples were washed four times to remove excess antibody.

Images of fluorescently labelled platelets were taken with AxioExaminer microscope (Carl Zeiss, Oberkochen, Germany) using a 40 × objective. At least 5 different fields of view were imaged in each well. Platelets exhibited a high variability in morphology and staining intensity between surfaces, which made classical thresholding based on signal intensity ineffective. Therefore, the machine learning based software Ilastik was used for platelet segmentation^28^. A pixel classification model was trained on 10 representative images. The model was used to generate probability maps of the analysed images. The maps were then thresholded at a probability value of 0.5 and quantified using the “Analyze Particles” feature of FIJI^29^. The Ilastik-based map generation and FIJI-based quantification were automated using Python scripts and FIJI macros^30^.

### Ninety-six-well aggregometry

Platelet aggregation in 96-well microplates was assessed according to the protocol of Vinholt et al.^31^, with minor modifications. Briefly, the wells of the microplates were filled with 10 µl of ADP and 90 µl sample (PRP or suspension of isolated platelets). ADP concentrations ranging from 0.1 µM to 40 µM were used to assess platelet aggregation, which was analysed using dose-response curve parameters. Control wells for PRP samples contained 10 µl saline and 90 µl PRP or PPP, while control wells for isolated platelet suspensions contained 10 µl saline and 90 µl platelet suspension or Tyrode’s buffer. Microplates were sealed with film and shaken at 1,200 rpm for 5 min at 37 °C. Absorbance was then measured using a VICTOR^™^ X4 microplate reader (Perkin Elmer, Waltham, MA, USA) at 595 nm.

### Statistical analysis

Results are expressed as arithmetic mean ± SD unless otherwise stated. Data for each assay were collected in five to eight independent experiments.

Data were tested for normality using Shapiro-Wilk test and for sphericity using Mauchly’s test. Differences between polymer surfaces were assessed by repeated measures analysis of variance (RM ANOVA) followed by Tukey’s post hoc test. The Greenhouse-Geisser correction was applied when the sphericity assumption was violated. The Friedman test followed by Dunn’s post hoc test for multiple comparisons were used when the normality assumption was not met. Student’s t-test or Wilcoxon signed-rank test were used to assess the effect of ADP on platelet deposition. The extra sum-of-squares F-test was used to assess differences between aggregation curves. The EC50 values for ADP were calculated from dose-response plots using non-linear regression analysis. Associations between variables were assessed using Spearman’s correlation coefficient. Statistical analysis was performed using the following software packages: Statistica v.13.3 and GraphPad Prism v.5.03 or v.8.0.1.

## Electronic supplementary material

Below is the link to the electronic supplementary material.

Supplementary Material 1

## Data Availability

The data that support the findings of this study (the results of area coverage quantification of adhered blood platelets, along with trained Ilastik model, ImageJ macros and Python scripts) are openly available in Zenodo repository at https://doi.org/10.5281/zenodo.14162001, reference number 14162000.
