# Supplementary material for: A non-binding surface demonstrates increased thrombogenicity under plasma-free conditions
Source: Sci Rep. 2025 Jul 2;15:23042. doi: 10.1038/s41598-025-06636-z (PMC12217689; doi:10.1038/s41598-025-06636-z)
Supplement: Supplementary file 1 — Supplementary Material 1 [file 41598_2025_6636_MOESM1_ESM.pdf]

A non-binding surface demonstrates increased thrombogenicity under plasma-free conditions

Cezary Watala, Boguslawa Luzak, Tomasz Przygodzki, Magdalena Boncler

Department of Haemostasis and Haemostatic Disorders, Chair of Biomedical Sciences, Medical University of Lodz, ul. Mazowiecka 6/8, 92-215 Lodz, Poland.

Supplementary Table S1. A brief overview of the study's main results.

**Method/Model**	**Platelets in plasma (PRP)**				**Platelets in buffer (PLT)**			
	**NB vs. PP**		**NB vs. PS**		**NB vs. PP**		**NB vs. PS**	
**Flow cytometry (CD62P and Fg binding):**	CD62P	Fg binding	CD62P	Fg binding	CD62P	Fg binding	CD62P	Fg binding
5-minute exposure, ADP 0	NS	NS	NS	NS	NS	NS	NS	NS
5-minute exposure, ADP 1 µM	NS	NS	NS	NS	NS	NS	NS	NS
5-minute exposure, ADP 5 µM	NS	NS	NS	NS	*	***	NS	**
5-minute exposure, ADP 10 µM	NS	NS	NS	NS	*	**	NS	NS
60-minute exposure, ADP 0	NS	NS	NS	NS	**	NS	**	NS
**Adhesion:**								
60-minute exposure, ADP 0	***		*		*		*	
60-minute exposure, ADP 10 µM	****		****		**		**	
**Microscopy:**								
5-minute exposure, ADP 0	***		**		**		**	
60-minute exposure, ADP 0	**		*		****		****	
**Aggregation:**								
5-minute exposure, ADP 0.1–40 µM	****		****		ND		ND	
**Secretion (sP-selectin):**								
60-minute exposure, ADP 0	NS		NS		NS		NS	

Statistically significant differences are indicated by asterisks (*P<0.05, **P<0.01, ***P<0.001, ****P<0.0001). Differences where NB<PP/PS are highlighted in green, and differences where NB>PP/PS are highlighted in red. Abbreviations: NB, non-binding microplate; PP, polypropylene; PS, polystyrene; Fg, fibrinogen; ND, not determined; NS, not significant.
